# Subnational benchmarking of health systems performance in Africa using health outcome and coverage indicators

**DOI:** 10.1186/s12916-015-0541-y

**Published:** 2015-12-14

**Authors:** Abdisalan Mohamed Noor

**Affiliations:** Nairobi Programme, Kenya Medical Research Institute/Wellcome Trust Research Programme, PO Box 43640-00100, Nairobi, Kenya; Information for Malaria (INFORM) Project, http://www.inform-malaria.org; Centre for Tropical Medicine and Global Health, Nuffield Department of Clinical Medicine, University of Oxford, CCVTM, Oxford, OX3 7LJ UK

**Keywords:** Assessment, Benchmarking, Child mortality, Health systems, Intervention coverage, Performance, Uganda

## Abstract

National health systems performance (HSP) assessments and benchmarking are critical to understanding how well the delivery of healthcare meets the needs of citizens. Benchmarking HSP has often been done between countries to inform the global public health space. However, its impact is likely to be far greater when implemented sub-nationally to inform actual decisions on resource allocations and performance improvements, especially in high disease burden, low-income countries, where the resource envelope available for health is inadequate. In their study, Roberts and colleagues assemble, analyse and map a minimum set of health intervention and outcome indicators from 1990–2011 to assess and benchmark HSP across the 11 regions of Uganda. This is the first empirical sub-national HSP benchmarking study in the country and the results have potentially important health system policy implications.

Please see related research: http://www.biomedcentral.com/1741-7015/13/285

## Background

Assessing and benchmarking health systems performance (HSP) is complex and often requires the inclusion of many indicators to develop a composite metric of performance [1]. The debate on the selection and interpretation of the appropriate indicators aside, in several African countries, where the disease burden is high and the resources available are inadequate, information systems are also weak and data on most indicators are not readily available [2]. This, sadly, is a story that has been repeatedly narrated, and a discussion on the way forward is beyond the scope of this commentary. The paradoxical ‘advantage’ of such a context with regard to benchmarking of HSP, however, is that African health systems focus on the delivery of a basic package of interventions [3], which could be monitored through an equally basic set of indicators. In as far as these interventions constitute the largest resource investments in health in these countries, it seems reasonable that measures of their population coverage and outcomes form the basis for HSP assessment and benchmarking.

Uganda is one the countries with the highest disease burden in Africa, with infectious diseases, respiratory infections, malaria and HIV/AIDS, compounded by high levels of malnutrition, as the main contributors to the burden of disease [4]. Information systems remain weak [5], and most data on health outcomes and intervention coverage are available as process data or collected through household surveys.

It is within this context that Roberts et al. [6] undertook their important study assessing the trends in child mortality and 20 maternal and health indicators related to child survival [7], computing the national and regional trends in all the indicators, and selecting a subset of 11 to benchmark the performance of the health system in each of 11 regions. They used established mixed methods approach [8] to estimate childhood mortality and implemented a Gaussian process regression, a non-parametric method for interpolating non-linear trends [4], within a Bayesian inference framework to predict trends in coverage indicators. To generate a composite measure of overall coverage, they computed the average coverage of the 11 indicators. Finally, linear correlations were estimated to measure the relationship between overall coverage and under-five mortality.

The analysis revealed mixed progress in Uganda. Nationally, under-five mortality has reduced by almost half, from 163 per 1000 live births in 1990 to 85 per 1000 in 2011, corresponding to increases in coverage of most health interventions. Interventions that required multiple contacts with the health system, such as the receipt of two doses of sulphadoxine pyrimethamine for the intermittent prevention of malaria during pregnancy and up to four antenatal care visits during pregnancy, remained largely unchanged.

Despite general progress, the study showed a marked and worrying regional disparity in child mortality and overall coverage as well as in some of the key component indicators. For example, Kampala had dramatically lower child mortality rates and a generally more favourable intervention coverage compared to the remaining regions. In contrast, the Karamoja region had a higher child mortality rate in 2011 relative to that in 1990, while estimates of underweight among children rose during this period. Additionally, in some regions, access to certain vaccines either declined or stagnated. All of the above suggest an inherent regional variability in HSP and describe the region-specific factors contributing to the disparity in health outcomes.

### Policy implications

The study by Roberts et al. [6] has immediate policy implications. For example, the Karamoja region, and potentially all those with a low overall coverage threshold and/or under-five mortality, should be prioritised for intervention and health system improvements by the government. Assessment of the trends in specific interventions and how these correlate with the prevalence of under-five mortality can highlight the priority actions to be implemented by policymakers. However, caution needs to be exercised as correlations or other associative relationships are not always indicative of causal relationships; nevertheless, the authors have selected interventions previously shown to have an impact on under-five mortality [7]. An overall increase in comprehensive access to antenatal care, intermittent prevention treatment in most regions, and childhood nutrition interventions in Karamoja, all require urgent action.

### Future directions and conclusions

The authors rightly conclude with a recommendation for continued tracking and analysis of subnational health trends to guide policy. They also list a number of study limitations, including the lack of data to analyse several important indicators, challenges of comparability of data between surveys, potential recall problems that may bias the measurement of some variables, and survey sampling deficiencies. Other limitations, however, are equally important and should be addressed in future studies. First, although the study approach and findings are highly relevant, the most recent estimates included correspond to 2011 and do not capture any changes in the 4 years since. This is likely due to the late release of the Uganda Malaria Indicator Survey 2014–2015 [9]. The delayed availability of important survey results that hinders timely policy decision-making is, unfortunately, a common and often unnecessary occurrence. Second, compared to broad measures of overall population coverage of the interventions, measures of effective coverage are more useful and combine the need for, use and quality of the interventions [10]. For example, in highly urbanised settings, such as Kampala, where intrinsic transmission is likely to be low, the efficacy of insecticide-treated nets against malaria infection is also likely to be low despite the high coverage, and would therefore achieve less impact compared to similar coverage levels in higher malaria transmission regions. The efficacy of insecticide-treated nets is also dependent on the level of insecticide concentration, which degrades with time, the physical condition of nets, and the levels of insecticide resistance. Most recent household surveys document net age and physical condition, although it is unlikely that this information is available in older surveys. It is also interesting to note that interventions for which coverage has stagnated over time are those requiring multiple contacts with the health system. This may suggest a general lack of access to these services, as well as issues such as inadequate and poorly distributed staff [11] and poor adherence to guidelines for disease treatment [12]. Inclusion of health workforce distribution per capita by region and data from health service provision assessments may provide useful indicators for service delivery and quality. Further disaggregation of the data to district level is likely to improve the practical utility of these results, accepting the degradation of estimate precision [13].

Finally, the Uganda study [6], as well as two recently published studies for Zambia [14] and Nigeria [15], is a commendable and relevant exercise at assembling and analysing important and available health metrics that are highly relevant to maternal and child health outcomes. With improvements, these studies will provide important policy guidance and should be implemented across sub-Saharan Africa.
